# Establishment of a guided, *in vivo*, multi-channel, abdominal, tissue imaging approach

**DOI:** 10.1038/s41598-020-65950-w

**Published:** 2020-06-08

**Authors:** Julia Bahlmann, Nodir Madrahimov, Fiene Daniel, David Theidel, Daphne E. DeTemple, Manuela Buettner, André Bleich, Axel Haverich, Alexander Heisterkamp, Stefan Kalies

**Affiliations:** 1 Institute of Quantum Optics, Leibniz University Hannover, Hannover, Germany; 2 Deutsches Zentrum für Lungenforschung e. V., Munich, Germany; 3 Lower Saxony Center for Biomedical Engineering, Implant Research and Development (NIFE), Hannover, Germany; 4 Department of Cardiac, Thoracic, Transplantation and Vascular Surgery (HTTG), Hannover Medical School, Hannover, Germany; 5 Department for General, Visceral and Transplant Surgery, Hannover Medical School, Hannover, Germany; 6 Institute for Laboratory Animal Science, Hannover Medical School, Hannover, Germany

**Keywords:** Endoscopy, Fluorescence imaging, Animal biotechnology

## Abstract

Novel tools in humane animal research should benefit the animal as well as the experimentally obtained data. Imaging technologies have proven to be versatile and also in accordance with the demands of the 3 R principle. However, most imaging technologies are either limited by the target organs, number of repetitive imaging sessions, or the maximal resolution. We present a technique-, which enables multicolor abdominal imaging on a tissue level. It is based on a small imaging fiber endoscope, which is guided by a second commercial endoscope. The imaging fiber endoscope allows the distinction of four different fluorescence channels. It has a size of less than 1 mm and can approximately resolve single cells. The imaging fiber was successfully tested on cells *in vitro*, excised organ tissue, and in mice *in vivo*. Combined with neural networks for image restauration, high quality images from various abdominal organs of interest were realized. The second endoscope ensured a precise placement of the imaging fiber *in vivo*. Our approach of guided tissue imaging *in vivo*, combined with neuronal networks for image restauration, permits the acquisition of fluorescence-microscope like images with minimal invasive surgery *in vivo*. Therefore, it is possible to extend our approach to repetitive imaging sessions. The cost below 30 thousand euros allows an establishment of this approach in various scenarios.

## Introduction

In human medicine, novel tools are a necessity to refine and improve surgical procedures or to pass new limits in imaging or detection of molecular compounds, leading to a benefit for the patient. Humane animal research is today defined by the 3 R principle: refinement, reduction, and replacement. This framework demands the same as above: Novel tools are necessary to improve animal condition and data output. Several imaging modalities allow repeated measurements in the same animal over a given time period. This longitudinal study design enables a reduction in animal experiments, as not as many animals have to be sacrificed than for endpoint based analysis methods as, for instance, for histologic analyses. Imaging methods applied in animal research include micro-computed tomography^1^, magnetic resonance imaging^2^, positron emission tomography^3^, ultrasound^4^, and optical imaging. Optical imaging has two significant advantages: Firstly, in combination with fluorescence, variety of different fluorophores such as dyes, antibodies, or fusion proteins are available^5,6^. These enable a high-contrast detection. Secondly, optical imaging typically reaches the highest resolution of all methods mentioned and can enable cell resolved studies.

Cellular resolution, combined with a longitudinal study design is, for example, mandatory for neuronal imaging or regeneration research^7^. This can be achieved using an imaging window combined with confocal or multiphoton microscopy^8^. Abdominal, cranial, mammary and skinfold imaging windows are described^9^. However, these windows have limitations: Due to its diameter only some organs can be placed adequately behind the window^10^. Furthermore, it is not possible to examine all abdominal or thoracic organs in one setup.

A different approach is the use of an endoscopic methodology^11–15^: Most commercially available endoscopes feature bright field illumination and detection, with limited resolution. However, also fluorescence imaging modalities have been developed and, for instance, been used in longitudinal studies of the intestine. The construction of a fiber-based cellular microscope can be realized using imaging fibers^16–20^, which contain several thousand fiber cores. These fiber optic probes with their high resolution have many benefits that can be used *in vivo*^21^: they can be placed via micromanipulators, allow repeated imaging for longitudinal studies due to their small size, and can be combined with fluorescence imaging. However, imaging is only possible on organ surfaces and the placement inside the animal is difficult. To visualize different tissues a guided method is necessary. Considering animal health and the intended possibility of longitudinal imaging, this method should also be minimally invasive. A good option to place and guide the fiber is using a second imaging modality, for example, ultrasound. However, ultrasound systems are also limited in resolution. An alternative would be to use a second endoscope. This would also facilitate orientation, as the organ of interest would be imaged in its native appearance.

**Figure 1:**
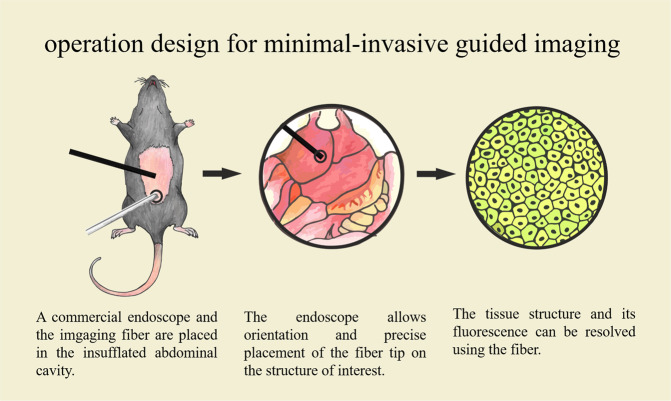
Overview of the design-concept of minimal-invasive guided imaging for humane animal research. A commercial endoscope is used for orientation and is combined with a self-built imaging fluorescence endoscope, which visualizes the tissue structure.

We have developed a double, guided, imaging approach using a combination of a commercially available endoscope, combined with a self-built fluorescence endoscope, which enables almost cellular resolution in four different fluorescence channels (see Fig. 1). The total cost of the combination of both systems is below 30 thousand euros, to make it achievable for the researcher and later for the practitioner. We demonstrate the applicability of our system *in vitro* and *in vivo* and compare it to fluorescence microscopy and confocal microscopy. Additionally, we investigated whether novel approaches such as neuronal networks can be applied to increase the image quality^22–26^. We envision that such an approach can be a very useful methodology in humane animal research, which might be later extended to human medicine.

## Methods

### Establishment of an optical setup for four-color fiber-based *in vivo* imaging

**Figure 2:**
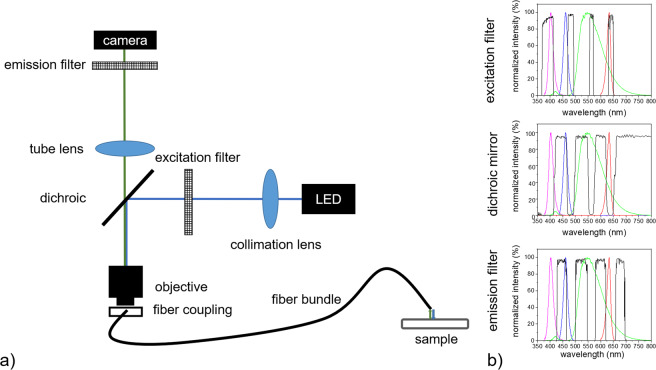
Schematic of the Four-color fiber-based *in vivo* imaging setup (**a**) and chosen filters for four-color imaging (**b**).

The fiber setup for imaging with near-cellular resolution consisted of a self-built fluorescence microscope, which can be built using optical shelf components, and an imaging fiber bundle. The used light source was a four channel LED (LED4D251, *Thorlabs GmbH*, Germany) with wavelength maxima at 405 nm, 470 nm, 565 nm and 625 nm. These wavelengths were chosen to enable the excitation of a wide number of fluorophores. This would allow the visualization of differently marked cells. To obtain a high signal to noise ratio, the collimated beam from the LED source was spectrally restricted via a four band band-pass filter (390/482/563/640 nm, *AHF analysentechnik AG*, Germany). A dichroic beam splitter (*AHF analysentechnik AG*) was applied to split excitation and emission light. The reflection maxima of the dichroic mirror (405/488/561/635 nm) were chosen to fit those of the LED source. A 20x objective lens with a numerical aperture of 0.5 served to bundle the beam for illumination of the proximal fiber bundle end. The fiber bundle (FIGH-30-850N, *Myriad Fiber Imaging Tech., Inc*., US) had a length of approximately 90 cm, 30.000 picture elements, a numerical aperture of 0.35, and a total diameter of about 950 µm. The emission light from the fiber was collected using the same objective and transmitted through the dichroic mirror. A 75 mm achromatic lens (*Thorlabs GmbH)* was used to focus the light on a fast CCD camera (340M-USB, *Thorlabs GmbH*), which can achieve up to 200 fps, and is crucial for *in vivo* imaging. Another four band band-pass emission filter (446/523/600/677 nm, *AHF analysentechnik AG*) spectrally separated the detected light at the camera (see Fig. 2).

### Physical and *in vitro* characterization of the four-color fiber-based *in vivo* imaging setup

The magnification, resolution, and spectral separation of the fiber-based setup were determined. The magnification of the setup was calculated with a standard stage graticule (GSQ2104, *Knight Optical, Ltd*., UK). An USAF resolution test chart (GRQ5001, *Knight Optical Ltd*.) was applied to determine the lateral resolution of the fiber probe. The spectral overlap was measured using a silver mirror for full back reflection of the excitation light. The maximum output power was determined with a powermeter (Newport Model 1919-R, *Spectra-Physics*, US).

*In vitro* characterization of the fiber was performed with HeLa cells. These were cultivated in DMEM medium supplemented with 10% fetal bovine serum, 100 U/mL penicillin/streptomycin (all reagents *PAN-Biotech GmbH*, Germany). The cells were stained with 2 µM Cacein-AM, 2 µM Hoechst 33342, and 0.5 µM MitoTracker Red (all reagents *Thermo Fisher Scientific Inc*., US) in culture medium for 20 min at 37 °C and 5% CO_2_ atmosphere to visualize cell metabolism, the cell nucleus, and mitochondria. In some experiments, cells expressing a mitochondria-tagged green fluorescent protein were used. The fiber-setup was compared to a commercial widefield fluorescence microscope equipped with an Andor Luca R EMCCD camera (*Andor Technology Ltd*., UK) for cellular imaging.

Additionally, the application of our fiber-based setup for imaging of tissue was analyzed and compared to widefield fluorescence and confocal microscopy. Organs were excised after final imaging (see below) and different dyes (2 mM rhodamine 6 G, 3 mM fluorescein, and 0.26 mM riboflavin; all dyes in 0.9% NaCl solution, all dyes *Sigma-Aldrich*, US) were topically applied. After a 10 min incubation period with the dyes, the organs were rinsed with NaCl solution and imaged with the fiber-based setup. Furthermore, organs of green fluorescent protein (GFP) expressing mice (C57BL/6-Tg(CAG-EGFP)1Osb/J) were imaged with the fiber based setup and compared to a commercial confocal microscope using the 488 nm laser line (Leica TCS-SP5, *Leica Microsystems*, Germany).

### Improving fiber-based imaging over neuronal network

**Figure 3:**
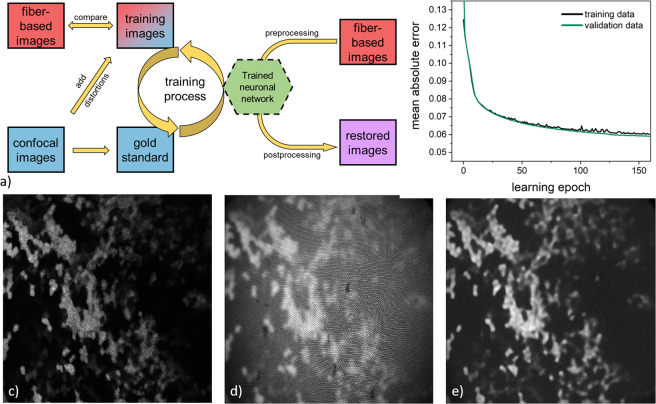
Illustrative scheme of the neuronal network processing. (**a**) Working flow for image restauration (**b**) Evaluation of the learning process: deviation of the mean error in the currently processed image from the gold standard image (see also supporting info). (**c**) Gold standard image of the liver using *ex vivo* confocal microscopy. (**d**) Modified training image. (**e**) Reconstructed training image.

Similar to widefield fluorescence microscopy, our fiber-based imaging approach is limited by blur from out-of-focus light. In microscopy, deconvolution is often applied to minimize this blur and restoration of the image. However, cross talk between neighboring fiber cores restrains its application in our scenario. To overcome this limitation, we decided to apply neural networks, trained with conventional microscopy data, for image restoration and improvement (see Fig. 3). Therefore, confocal images of the organs of interest were acquired. These were used as gold standard input images for the content-aware image restoration toolbox CSBDeep [21]. This toolbox is based on convolutional neural networks. Briefly, the training images were produced by altering the gold standard images via blur, noise, and intensity decrease. Additionally, we added the fiber core structure to these images (see supporting information for all details). During the training process 20% of the input was used as validation data. The network training was verified based on the evaluation of the training history to prevent potential under- or overfitting. Therefore, the network performance was measured on the training and the validation data by calculating the mean absolute error (see Fig. 3). The history consists of data, which represents the difference between reconstructed training image and gold-standard image in respect to the training time. Finally, the network was applied to the images. All details on the training and processing are available in the supporting information file.

### Establishment of the guided surgical procedure

To establish the operation technique, seven female C57BL/6J mice were used. For visualization of the tissue of interest, RhodaminG6 (1 mM) and FITC-Dextran (10 mg/ml) were intravenously injected. Furthermore two male and one female C57BL/6-Tg(CAG-EGFP)1Osb/J mice were used. In these mice, an intravenous injection of dyes was not necessary, due to the native expression of the green fluorescent protein (GFP). All mice were between 70 and 160 days old and they were bred in the laboratory animal science of the Medical School Hannover.

To establish the operation technique, all mice were anesthetized with 5% isoflurane. During the surgical procedure 1.75–2.5% isoflurane was used. We injected butorphanol (5 mg/kg) subcutaneously as an analgesic. The mice were placed on a heating plate (PHYSIOLOGICAL MONITORING SYSTEM, *Havard Apparatus*, US) and monitored in temperature, heart rate, and respiration. All mice were sacrificed and dissected after operation for investigation of potential injuries set during the procedure. The guided surgical procedure was established as follows: First, the abdominal cavity was insufflate with a maximal pressure of 5 mmHg CO_2_ using a 1 mm silicon tube and a venous catheter of 14 G. A commercial endoscope with a 30° angle and a diameter of 1.9 mm (30421901, *Eickemeyer*, Germany) was inserted at the *linea alba* using a 10 G needle. After an initial exploration of the abdominal cavity, the fiber bundle from the four-color fiber-based *in vivo* imaging setup was placed with a trocar (58717 R, *Karl Storz*, Germany) through the *linea alba* at a distance of about 1 cm *cranial* to the first endoscope. This allowed to precisely guide the fiber bundle via direct visualization with the first endoscope and to place it on the organ of interest. A combination of manipulation arms (M11, *wpiinc*, US and Nr. 1040128, *Karl Heck GmbH*, Germany) and a micromanipulator (*Märzhäuser*, Germany) were used for precise placement of both endoscopes.

## Results

### Characterization and application of the fiber-based probe *in vitro*

Before the *in vivo* application of the fiber-based imaging setup, it was characterized *in vitro*. The output power was measured at the fiber tip and calculated based on the fiber tip surface area (see Table 1). The spectral noise or bleed through was defined as the amount of back reflected light at highest output power, reaching the CCD, relative to the total dynamic range of the CCD during full reflection. The same parameters were used as during an imaging session (See Table 1). The lateral resolution of the self-built setup was experimentally determined to be 3.1 μm.

**Table 1:** Characterization of the fiber-based setup detecting the maximal output power and the spectral noise.

Central LED wavelength [nm]	Maximum output power [mW/cm²]	Fluorescence noise at an exposure time of 20 ms [%]
405	282	19.7
470	123	0.6
565	88	0.5
625	352	1.1

The four LED spectra were examined individually and the LED power was measured at the fiber tip. Maximum output power was calculated based on the area of the fiber tip.

**Figure 4:**
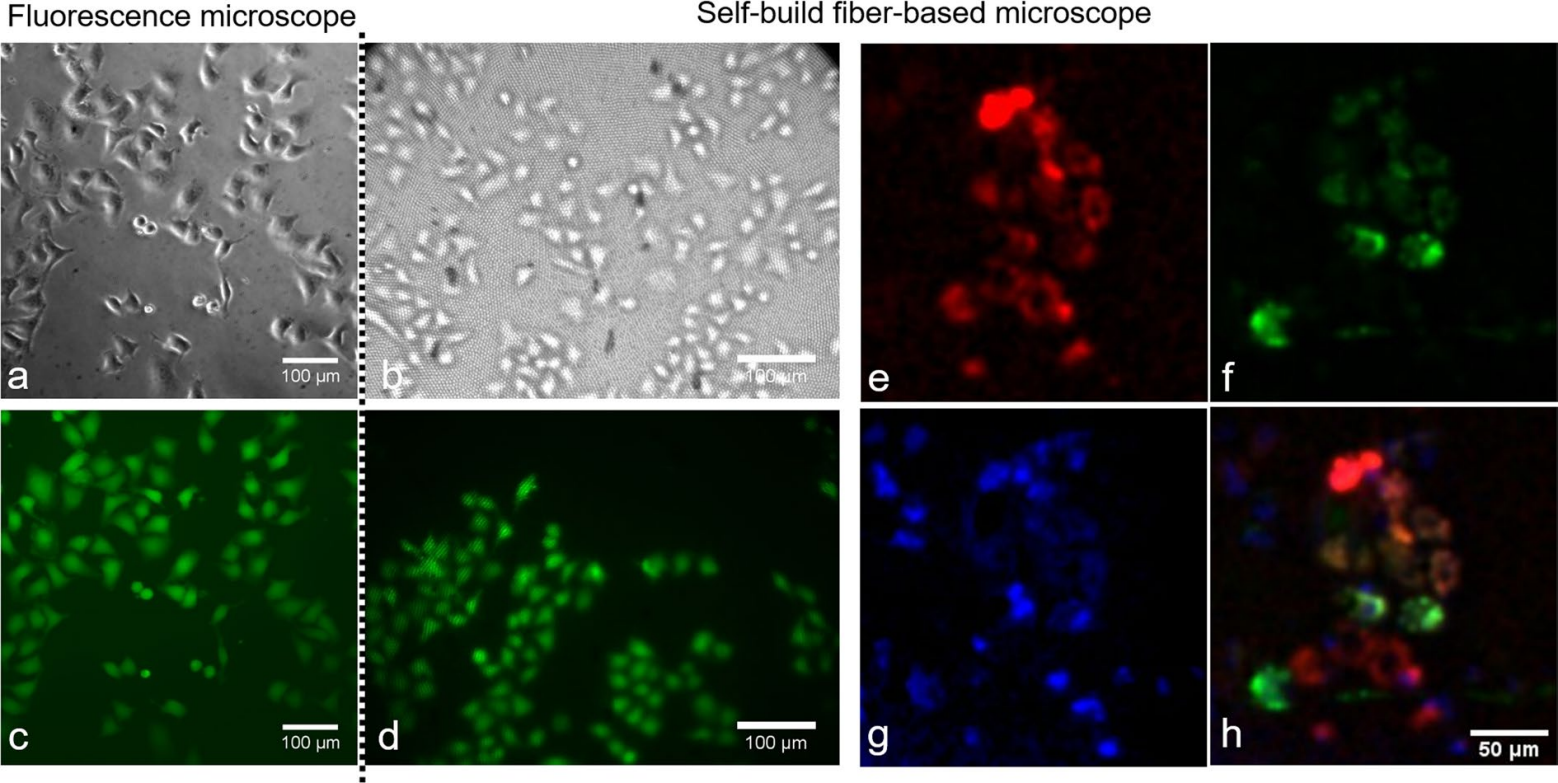
Brightfield and fluorescence microscopic images using a commercial fluorescence microscope (**a,c**) and our fiber- based setup (**b,d**) in Calcein stained cells. Demonstration of fluorescence imaging of three different dyes: MitoTracker-Red (**e**), mitochondria-tagged GFP (**f**) and Hoechst 33342 (**g**), as well as an overlay image of all channels (**h**).

Next, the application of the fiber-setup in cultured cells was tested. Single cells could be well-resolved using bright field illumination with a flash light. Calcein AM stained cells were compared to a commercial fluorescence microscope (see Fig. 4). Staining of the cells with Hoechst 33342 (excitation channel 405 nm), mitochondria-tagged GFP (excitation channel 470 nm) and MitoTracker Red (excitation channel 565 nm) was applied to test the fluorescence imaging capabilities. All three dyes could be excited with minimal overlap. The overlap was caused by the broad excitation spectrum of MitoTracker Red.

**Figure 5:**
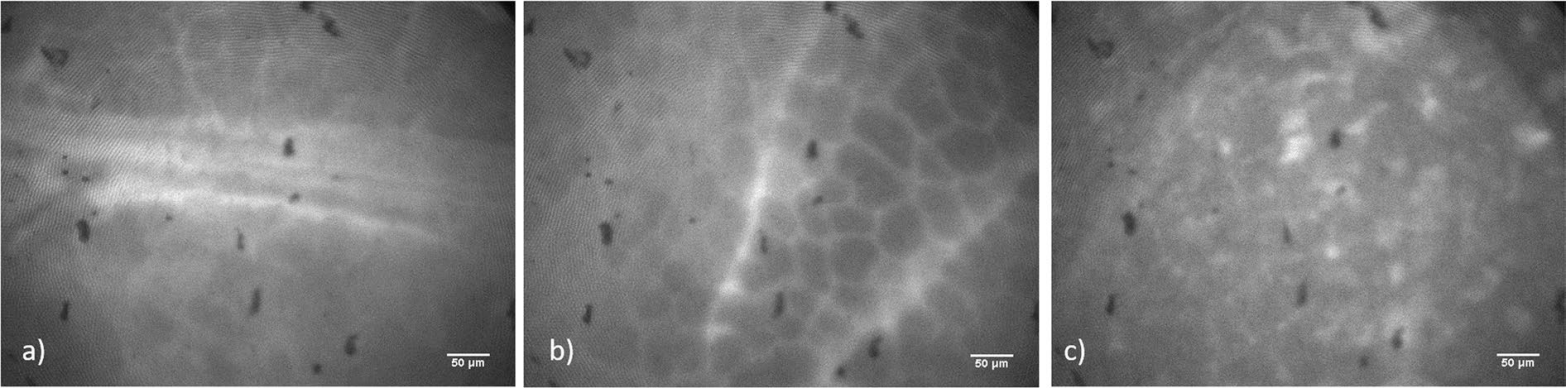
Fiber-based imaging after topical staining of pancreas *ex vivo* using fluorescein (**a**), riboflavin (**b**) and rhodamine 6 G (**c**).

Furthermore, the fiber-setup was tested on excised tissue. The organs were stained with rhodamine 6 G (excitation channel 470 nm), fluorescein (excitation channel 470 nm), and riboflavin (excitation channel 470 nm) (see Fig. 5). The rhodamine staining did not work as well as the other dyes.

**Figure 6:**
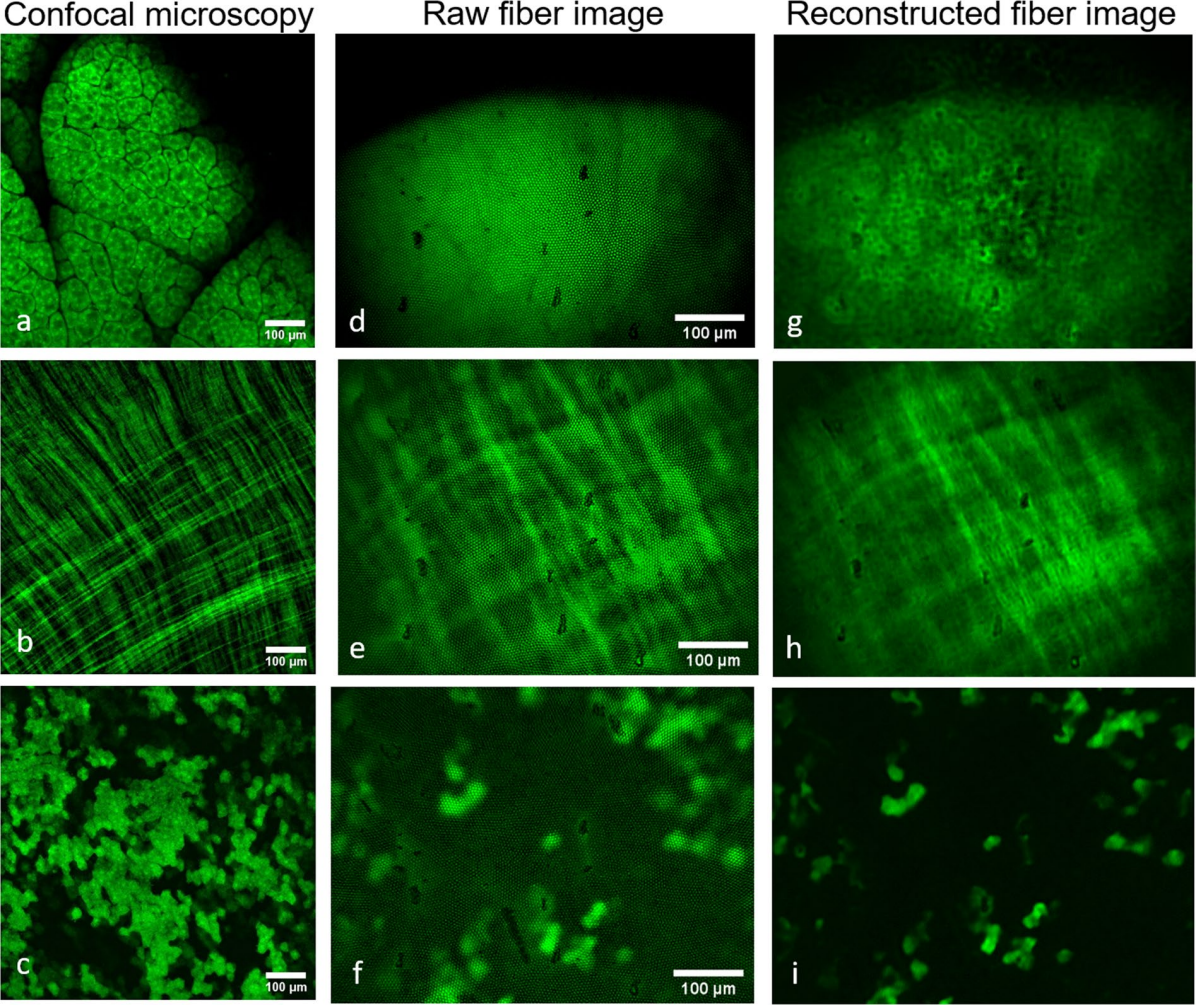
Imaging *ex vivo* organs of GFP-expressing mice using our fiber-based setup (**d–f**) and a confocal microscope (**a–c**). Compared to the fiber-based images, which were reconstructed using neuronal networks (**g–i**). For comparison, pancreas (**a,d,g**), jejunum (**b,e,h**) and liver (**c,f,i**) are shown. All images were acquired independently and do not depict the exact same place.

Next, the fiber–based setup was tested on excised tissue of GFP expressing mice to visualize characteristic structures of the organs. For comparison, theses organs were also analyzed with a confocal microscope (see Fig. 6). Compared to confocal microscopy, a blur was perceivable in the fiber-setup images. However, tissue and organ structure were visualized well and could be identified and analyzed in both, the confocal and the fiber-setup.

### Application of neural networks for image improvement

To improve the imaging results and facilitate the evaluation of the images, we applied the content-aware image restoration toolbox CSBDeep [21]. It was trained with confocal images for different organs from the GFP-expressing mice. Therefore, we altered our confocal images via addition of the fiber cores, blur, noise, and intensity decrease (please see Supporting Information – Fig. 1). Initially, a reconstruction without fiber cores was performed. However, addition of the cores to the training data proved to be more robust Therefore, we altered our confocal images via addition of the fiber cores, blur, noise, and intensity decrease (please see Supporting Information – Fig. 2). In case of the pancreas, 140 training images were used, for the liver we used 124 images, and 131 images for the jejunum. To obtain the best reconstruction, the initial learning rate, network depth, and number of training epochs and training steps per epoch were varied. The reconstruction of the training data proved to be reliable for all organs. After reconstruction, the structure of the liver could be well identified after the application of the neuronal network (see Fig. 6). The reconstruction of the pancreas was less successful, while the jejunum showed the worst reconstruction. This is probably caused by the elongated muscle structure of the jejunum^26^.

### Characterization and application of the fiber-based probe *in vivo*

**Figure 7:**
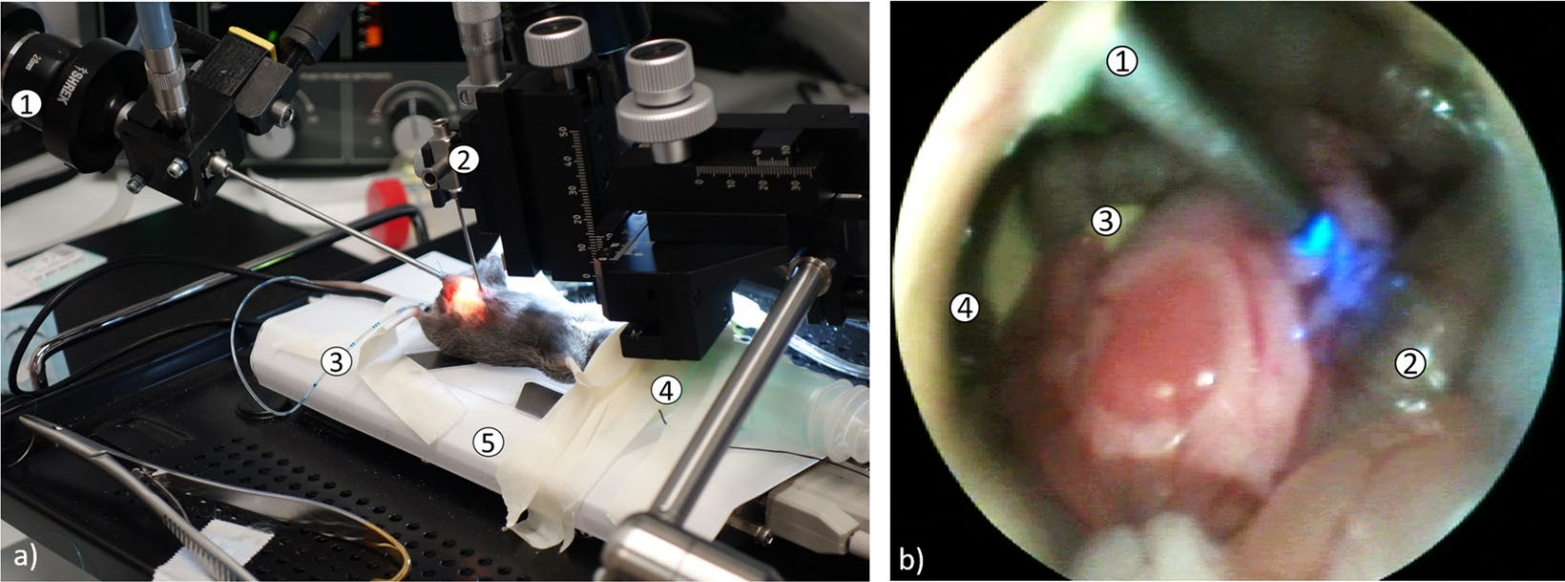
(**a**) Photograph of the surgical procedure: 1. commercial endoscope, 2. micromanipulator with the trocar for the fiber bundle, 3. insufflation-pipe, 4. isoflurane-mask, 5. heating-plate. (**b**) Endoscopic view through the commercial endoscope during the imaging procedure *in vivo*: 1. trocar for the fiber-optic with fiber bundle and blue illumination, 2. intestinal loop, 3. pancreas, 4. liver.

**Figure 8:**
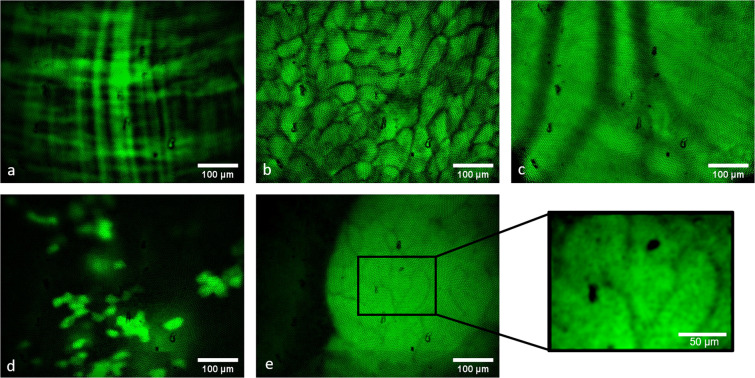
Fiber-based endoscopic *in vivo* visualization of (**a**) jejunum, (**b**) fat, (**c**) blood vessel in the abdominal muscle, (**d**) liver, (**e**) pancreas with zoom-in on detailed structure in GFP-expressing mice. For the zoom-in image, a gaussian-blur filter was applied to remove the structure of the fiber cores.

After the characterization of the setup, we tested the *in vivo* application (see Fig. 7). Therefore, a surgical procedure was established and tested in two different mouse strains. The abdominal cavity was explored and the organs were macroscopically examined. Next, the fiber-bundle was inserted and placed on different organs like liver, pancreas, and bowel segments. Placing the fiber-bundle on organs deep in the abdominal cavity is challenging. Moving the fiber-bundle with a micromanipulator showed up to be very precise. The fiber could be placed, for example, on different parts of the liver lobes. By injecting rhodamine G6 and FITC, the cells could be visualized. Compared to the excised organs, the tissue structure could be well identified. However, the injected dyes did not always distribute perfectly. Using the GFP expressing mice breed, we observed no differences to the excised organs (see Fig. 8). During the dissection, no major injury could be found. However, the trocar used for the fiber-bundle might cause slight hematomas.

## Discussion

Humane animal research requires novel methodologies, such as specialized imaging techniques. We developed a double imaging approach. We used a self-built fiber-bundle-based fluorescence endoscope guided by a commercially available endoscope, to achieve resolution on cell level *in vivo*.

Compared to other earlier fiber-based setups^19,21^, we designed our self-built fiber-based endoscope for a multicolor approach using off-the-shelf components and a material cost of below 30 thousand euros. Multicolor imaging enables the identification of different tagged cells in one imaging session. We have shown that different dyes can be used with our setup. By selecting the appropriate LED for excitation, many fluorophores can be excited in the same imaging session. The power of excitation light and therefore the excitation efficiency of our setup is similar to a commercial widefield fluorescence microscope, the lateral resolution of our setup is equivalent. *In vivo*, the application of our setup is complicated by animal movement due to breathing and the three dimensional organ structure. To overcome this problem, we used neuronal networks for image improvement, which were trained with confocal images. This proved to be a robust and easily applicable approach. It can also help to overcome optical aberrations in the setup, such as spherical aberrations^27^, which might have been caused by the emission filter placed in front of the camera in our setup. Currently, the processing was only applied on one imaging channel, but it can be extended to different channels in further studies.

Unfortunately, it is currently not possible to acquire three dimensional z-stacks with our setup or to visualize cell structures in deeper layers. Recently, Orth *et al*. demonstrated the feasibility of depth visualization in a single exposure by analyzing the transmitted light field through a fiber-bundle^28^. This would allow to visualized tissue down to approximately 80 µm depth with the option of refocusing.

A major difficulty in fiber based imaging is the exact placement of the fiber *in vivo*. In particular longitudinal studies require the possibility of certain relocalization for imaging the same region of interest in successive imaging sessions.

We used an endoscopic guiding system to place the fiber-bundle and to visualize different organs in the same mouse. The commercial endoscope proved to be a reliable tool to guide the fiber optic. We were able to explore the abdominal cavity and precisely place the fiber on different organs, for example liver, pancreas, and different bowel segments. Using the fiber-based microscope, we were able to visualize tissue structures on the surfaces of these organs in mice. This was possible after intravenous application of dye and when analyzing in GFP expressing mice. To place the fiber on organs deeper in the abdominal cavity, additional surgical steps would have to be taken into account, such as an endoscopic manipulator to move tissue. In contrast to other longitudinal approaches, for example, the abdominal imaging window^8^, our setup is nevertheless more flexible. The low diameter of both, the fiber and the endoscope, also enables a minimal-invasive surgical procedure.

## Conclusion

We have demonstrated that a guided, fiber-based setup could be a beneficial tool in animal research. To determine the limitations of the setup, we will compare healthy organs to diseased ones. A longitudinal study design will follow in order to examine wound healing and complications caused by repeated surgical procedures. This will hopefully enable a decrease of animals used in research by improving the data output in each individual animal. Due to the minimal-invasive character, the fiber-based setup is also a refinement and thereby another step towards humane animal research.

## Declaration

### Ethics approval and consent to participate

The experiments were in accordance with the German Animal Welfare Legislation and approved by the local Institutional Animal Care and Research Advisory Committee and permitted by the Lower Saxony State Office for Consumer Protection and Food Safety (reference number 18/2971).

## Supplementary information

supplementary info.

## Data Availability

The datasets during and/or analyzed during the current study are available from the corresponding authors on reasonable request.
