# Supplementary material for: Establishment of a guided, in vivo, multi-channel, abdominal, tissue imaging approach
Source: Sci Rep. 2020 Jun 8;10:9224. doi: 10.1038/s41598-020-65950-w (PMC7280182; doi:10.1038/s41598-020-65950-w)
Supplement: Supplementary file 1 — supplementary info. [file 41598_2020_65950_MOESM1_ESM.pdf]

Supplementary Information to:

Establishment of a guided, in vivo, multi-channel, abdominal, tissue imaging approach

Julia Bahlmann[1,2,3,*], Nodir Madrahimov[2,4], Fiene Daniel[1,3], David Theidel[1,3], Daphne E. DeTemple[1,3,5], Manuela Buettner[6], André Bleich[6], Axel Haverich[2,4], Alexander Heisterkamp[1,2,3,#], Stefan Kalies[1,2,3,*,#]

[1]*Institute of Quantum Optics, Leibniz University Hannover, Hannover, Germany*

[2]*Deutsches Zentrum für Lungenforschung e. V., Germany*

[3]*Lower Saxony Center for Biomedical Engineering, Implant Research and Development (NIFE), Hannover, Germany*

[4]*Department of Cardiac, Thoracic, Transplantation and Vascular Surgery (HTTG), Hannover Medical School, Hannover, Germany*

[5]*Department for General, Visceral and Transplant Surgery, Hannover Medical School*

[6]*Institute for Laboratory Animal Science, Hannover Medical School, Hannover, Germany*

Training and reconstruction procedure for the application of CSBDeep

We included the python library provided by the CSBDeep [1] project in a custom python script. The library includes a deep neural network structure called U-Net, primarily specialized in enhancing images from fluorescence microscopes. We trained this network structure on images of the pancreas, jejunum, and liver separately. For the pancreas, we used 140 gold-standard images from which we generated 420 training images. The network specialized on images from the liver was trained with 124 gold-standard images and 372 training images. The model for the jejunum was calculated by using 131 gold-standard images and 393 training images. All images were saved as TIFF files with a size of 512 x 512 pixels and processed as gray-scale images.

To generate training images, which resemble the image quality of the endoscope, we took confocal images of the different organs as gold-standard and modified them digitally to obtain the training images. We added different levels of blur and noise to the training images and varied the image intensity (see and **Supplementary Table 1** and **Supplementary Figure 1**).

**image operation**	**range**
intensity variation	0.1 – 30
convolution	sigma range 3 - 7
gaussian poisson noise	true

Supplementary Table 2 Image operations and applied range to generate training data.

Following we compared three different approaches for training of the neuronal network. First, training was performed on simply blurred images ("blurred" network approach). This was used in combination with final images, which underwent a gaussian blur to remove the fiber core structure before reconstruction. Second, training was performed with synthetic fiber cores to directly reconstruct fiber images ("synthetic fiber" network approach). We used the diameter of the fiber cores of the endoscope to add a layer of adjacent circles with the same diameter to the training images. This layer was blurred after addition, to merge the synthetic core structure with the training image. Third, we created an overlay image of the fiber acquired with brightfield illumination from our setup and the training images ("overlay fiber" network approach). All three approaches were analyzed and compared.

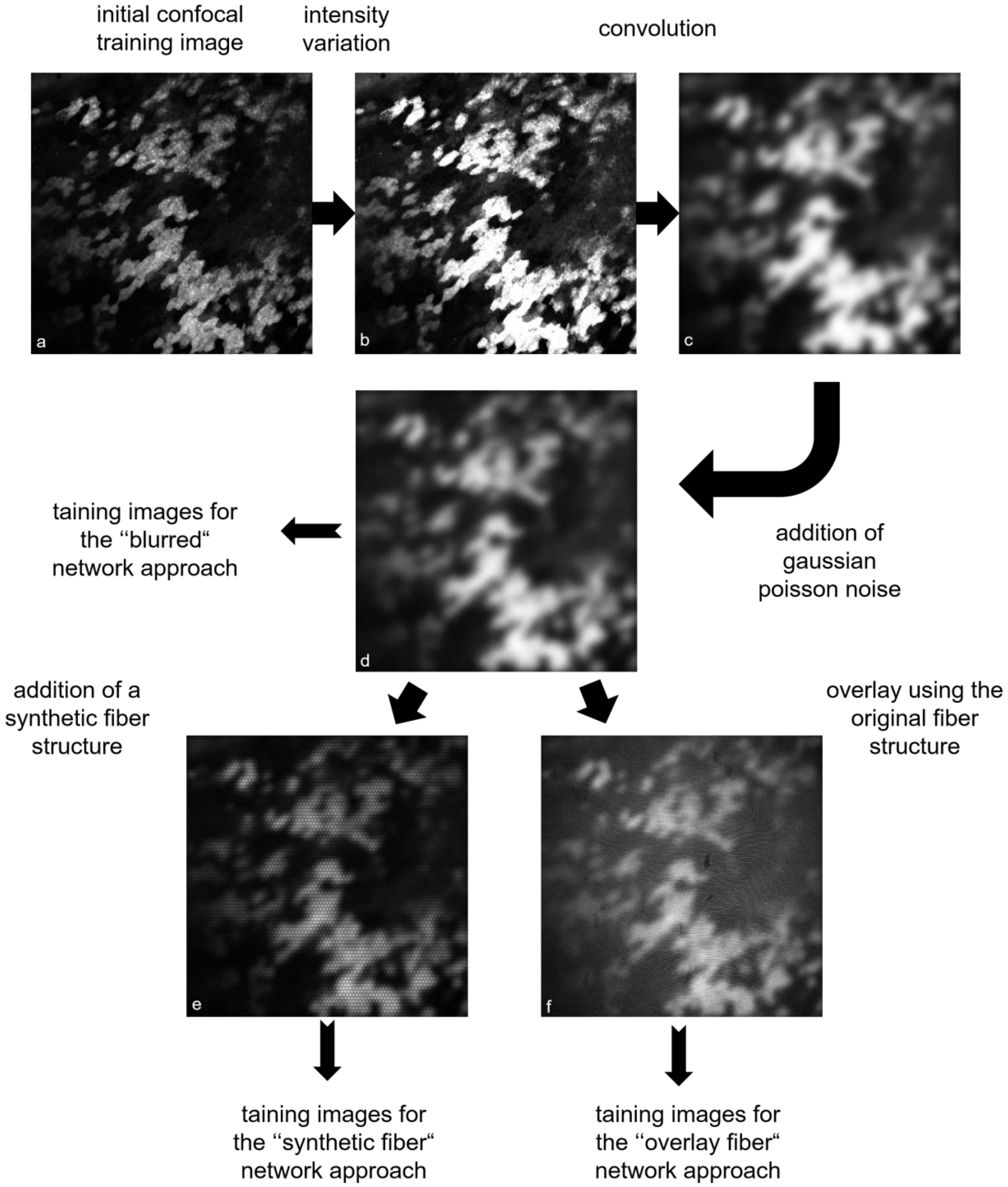

Supplementary Figure 3 Generation of training data exemplarily shown for a liver image. Initially, we used three different training approaches. The “blurred” approach was later only applied to unknown images, which were also blurred to remove the fiber cores. The “synthetic fiber” and overlay fiber” approach were used for direct reconstruction of fiber images.

Training of the neuronal networks and reconstruction of images

Each data set was split into a training set and a validation set. We used 80 % of the data as training set and 20 % as validation set, which is a common approach in deep learning [2]. Training accuracy was measured by the mean absolute error to the gold-standard image. Training was performed on a NVIDIA GTX 1060 GPU and took 3h33m for the liver data, 2h for the jejunum data set and 2h13m for the pancreas data set ("blended fiber approach"). For the application of the trained models we used the CSBDeep plugin for Fiji.

As detailed above, initially, we removed the fiber core structure in the data set by applying a gaussian blur to the images ("blurred" network approach). The networks were then trained using these images. Before applying the trained network to the unknown images, we also blurred these images. However, the reconstructed images showed many artifacts (see **Supplementary Figure 2**). Blurring of the images does not lead to a complete loss of the core structure even though the core structure is not visible for the eye. Therefore, blurring the images beforehand is not recommendable, instead we tried to remove the core structure using the network.

We decided to keep the core structure and to increase the depth of the U-Net architecture by resembling the fact, that another geometric structure has to be learned by the network. The network trained on the images with an added "synthetic fiber" layer, converged during the training process (see Supplementary Figure 2) with a training time of 3h29m. Restoration on the training images worked well. But applying the network to unknown images showed, that the original fiber structure could not be removed completely. Especially areas with potentially damaged fiber cores showed artifacts. Additionally, the fluorescent background light is not removed, resulting in comparatively low contrast.

The network with the "blended fiber" approach showed the best result. In the training history with a training time of 3h33m, the generalization error lies just below the training error. Because the gap between training and generalization error stays the same with increasing capacity of the model, the model is still converging. The mean absolute error of the network is the highest for all approaches with a difference of roughly 0.01 to the other models. This is not surprising, because the training set is the most challenging. Using an image of the actual fiber in training resulted in the best reconstruction. Areas with damaged fiber cores showed little artifacts and the fiber core structure was removed completely in the relevant areas.

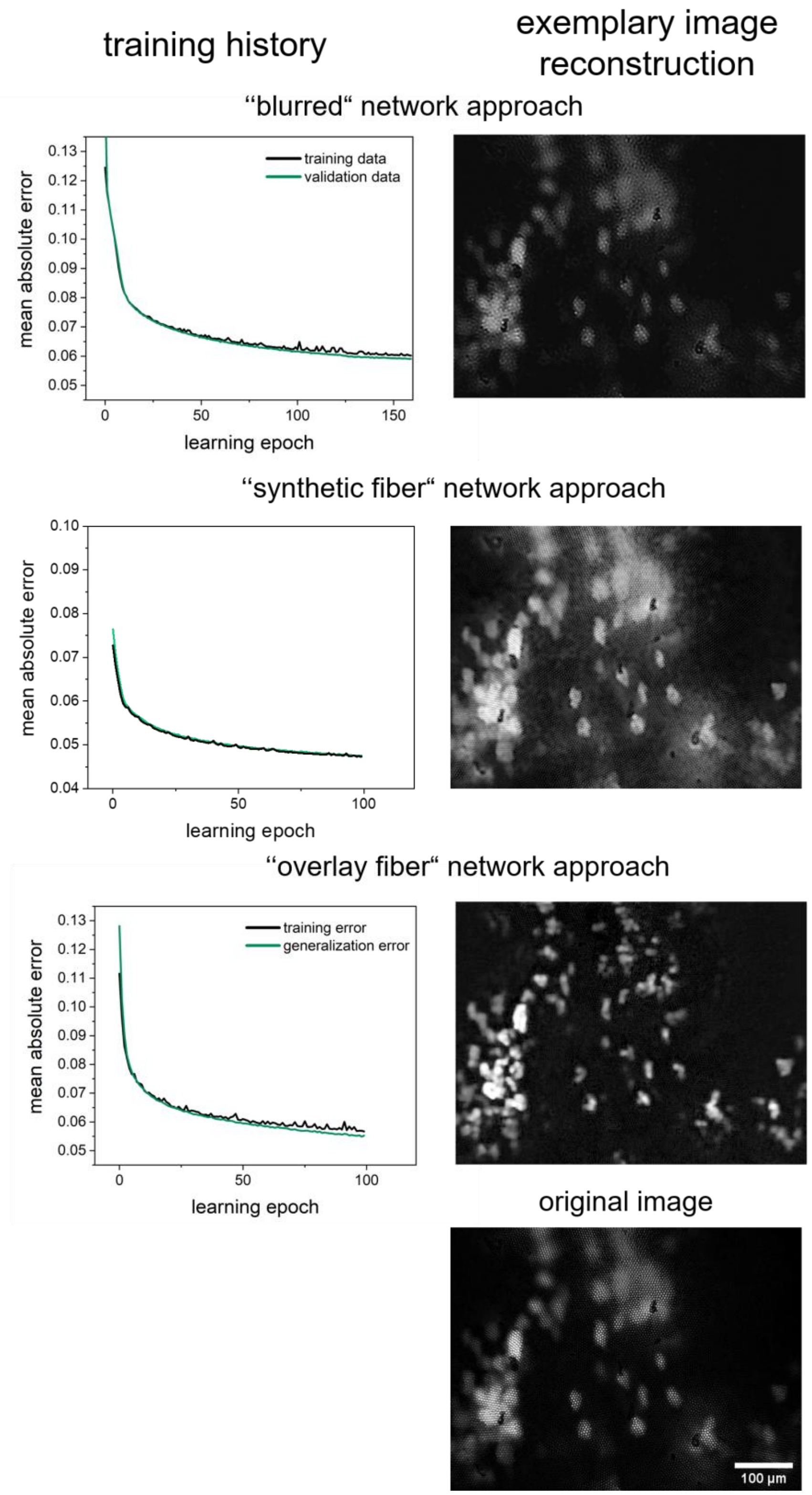

Supplementary Figure 2 Network training history and exemplary image reconstruction of an unknown image. The models appear to be converged for each approach. The resulting mean absolute error of the “overlay fiber” approach is the highest because the data set is the most challenging. The quality of restoration differs noticeably. The third approach has the most sophisticated reconstruction.

Supplementary Literature

1. Weigert M, Schmidt U, Boothe T, Müller A, Dibrov A, Jain A, et al. Content-aware image restoration: pushing the limits of fluorescence microscopy. Nature Publishing Group; 2018;15:1090–7.

2. Goodfellow I, Bengio Y, Aaron C. Deep Learning. MIT Press; 2016.
